# A Cross-Sectional Study of Glycemic Status and Zinc Level in Patients with Beta-Thalassemia Major

**Published:** 2017-10-01

**Authors:** Mohammad Ali Mashhadi, Zahra Sepehri, Zahra Heidari, Mahmoud Ali Kaykhaei, Aliyeh Sargazi, Farhad Kohan, Hanieh Heidari

**Affiliations:** 1 Hematology/Oncology Department, Zahedan University of Medical Sciences, Zahedan, Iran; 2 Internal Medicine Department, Zabol University of Medical Sciences, Zabol, Iran; 3 Endocrinology Department, Zahedan University of Medical Sciences, Zahedan, Iran; 4 Student Research Committee, Zabol University of Medical Sciences, Zabol, Iran; 5 Zahedan University of Medical Sciences, Zahedan, Iran

**Keywords:** Beta thalassemia, Diabetes mellitus, Zinc

## Abstract

**Background:** Endocrinopathies and diabetes mellitus are prevalent in patients with beta-thalassemia major

Recently some studies demonstrate a link between low levels of serum zinc level and higher prevalence of diabetes. The aim of this study was to evaluate the glucose tolerance in patients suffered from beta-thalassemia major and determine the association of Homeostasis Model Assessment (HOMA) parameters with zinc status among these patients.

**Materials and** **Methods:** In this cross sectional study, clinical data of patients who were suffered from thalassemia major, aged≥10 years were collected. Serum ferritin concentration, fasting blood sugar, fasting blood insulin and serum zinc level were assessed after overnight fasting. Moreover, oral glucose tolerance test was performed. Homeostasis Model Assessment (HOMA-2) was used for calculating beta-cell function, insulin resistance and sensitivity for normoglycemic and pre-diabetic subjects.

**Results:** of the 163 patients diagnosed with beta-thalassemia major, 10%, 53% and 37% were diabetic, pre-diabetic and normal, respectively. Mean serum zinc concentration was equal to 18.90±10.93µg/dl, and it was not significantly different across diabetic, pre-diabetic and normal groups. Pre-diabetic patients had significantly lower beta-cell function compared to normal subjects (P=0.0001). An inverse relation was documented between beta-cell function on one hand and total units of blood transfusion and ferritin level on the other hand (r=-0.29, P=0.004 and r=-0.27, P=0.03, respectively). The analysis adjusted for multiple possible confounders showed that there is no significant association between HOMA parameters and serum zinc level.

**Conclusion:** Impaired glucose metabolism and low serum zinc level were quite common among our study participants. The findings of the study also signifies the substantial role of follow-up in early detection and appropriate treatment.

## Introduction

 Β-thalassemia major is a hereditary disorder which suppresses the production of globin beta chains. This condition results in severe anemia. B-thalassemia affects a significant population in Mediterranean countries, India and Southeast Asia^1^. Patient’s life expectancy and survival are critically dependent on blood transfusion, but iron overload is an unexpected side effect which frequently causes lesions in heart, liver and endocrine glands; therefore diabetes mellitus in these patients is a considerable complication with significant morbidity and mortality^2^^-^^6^. The prevalence of patients who are suffered from both diabetes mellitus and thalassemia major is 2.3-26%^5^^,^^7^^-^^11^. Although it is assumed that the principal mechanism of diabetes mellitus is langerhans islets haemochromatosis which results in insulin deficiency, more complicated mechanisms are interfered in its pathogenesis such as insulin resistance, impaired hepatic metabolism of glucose and insulin due to iron overload, abnormal homeostasis of trace elements, genetic factors and family history of diabetes mellitus^11^^,^^12^. There are plenty of studies on humans and animals that demonstrate a link between low levels of zinc and higher prevalence of diabetes. Zinc is an essential element for insulin synthesis, storage and secretion. Just like insulin, zinc maintains the structure of some antioxidant enzymes such as superoxide dismutase; thus zinc as an antioxidant inhibits insulin resistance^13^^-^^15^. 

In this cross-sectional study, we tried to evaluate the prevalence of diabetic and pre-diabetic patients with impaired glucose tolerance among beta- thalassemia major patients. A further aim was to evaluate any possible association between serum zinc level and beta-cell function, insulin resistance, insulin sensitivity and related impaired glucose tolerance in beta-thalassemia major patients.

## MATERIALS AND METHODS

**Study design and population**

This cross-sectional study has been conducted on patients at the thalassemia clinic in Ali-Asghar Hospital, Zahedan, Iran. The study protocol was approved by the Local Medical Ethics Committee. Written consent was provided and agreement obtained from patients, patients’ parents or patients’ legal guardians in all cases. This has been done with the declaration of Helsinki. Patients aged more than 10 years with thalassemia major were enrolled in this study. All of them received regular blood transfusion with an interval of 4 to 5 weeks. The purpose of blood transfusion is to maintain hemoglobin at least 9.5 gr/dl before each transfusion. Besides, Desferoxamine is the iron chelating agent. It is used depending on serum ferritin level at a dose of 40-50 mg/kg for at least five days each week. 

**Evaluations**

After enrollment, all patients underwent assessment, including demographic data, past medical and surgical history, age at the first blood transfusion time, age at the initiation of therapy with chelating agent, duration of iron chelation therapy, physical examination and medication usage. One supervisor processed all data collection. Patients’ weight with minimal dressing and without shoes was measured, using digital weight machine with measurement error of less than 100g. Patients’ height was measured without shoes and with normal state of shoulders using a tape measure.

Blood sample was collected at 8:00 am after 8 hours of fasting for the purpose of fasting plasma glucose measurement. Afterwards, 75 gram of oral glucose administered and next blood sample obtained 2 hours later. 

Glucose oxidase technique was used for serum glucose measurement (Pars Azmun, Iran) and solid-phase competitive chemiluminescent enzyme immunoassay was used for serum insulin level definition (Diagnostic Product LIAISON, Italy). All commercial kits had been used previously with an intra-assay and inter-assay coefficient of variation of 5.0% and 8.0%, respectively. Ferritin was measured by a two-site immunoluminometric assay (Byk-Sangtec Diagnostica; Dietzenbach, Germany). Serum zinc level was measured using atomic absorption spectrophotometry (Spectr AA 240fs, 2009, USA).

Consistent with American Diabetese Association (ADA) guidelines, impaired glucose tolerance in pre-diabetic cases was diagnosed if 2h post-glucose blood sugar was in the range of 140-200 mg/dl and impaired fasting glucose was defined as a fasting blood sugar level of 100-125 mg/dl.

Diabetic cases required one of these three conditions: 1) Elevated fasting plasma glucose≥126mg/dl; 2) plasma glucose≥200 mg/dl after oral glucose uptake; and 3) at least one symptom related to diabetes (polydipsia, polyuria and weight loss) and random plasma glucose≥200mg/dl. Treatment with insulin or oral hypoglycemic medication was considered as diabetes.

Homeostasis Model Assessment (HOMA2) was used for calculating beta-cell function (%B), insulin sensitivity (%S) and insulin resistance (IR) for normoglycemic and pre-diabetic subjects. The input data were entered into HOMA2 calculator to measure serum insulin and fasting blood sugar.

(University of Oxford, website; http://www.dtu.ox.ac.uk/homacalculator/index.php).

**Statistical analysis**

Analysis was performed using statistical software package (STATA, STATA Corporation, Texas, USA) version 11.0. Continuous variables were presented as mean values and standard deviation as well as categorical variables were presented as absolute number and percentage. Categorical variables were analyzed using chi-square test and continuous variables were analyzed using analysis of variance test (ANOVA). Non-parametric tests were used for analyzing data that did not follow a normal distribution. The correlation between Zinc level as a continuous variable and other parameters was assessed using Pearson’s rank correlation analysis. Simple and multiple linear regressions between zinc and glycemic status were analyzed. Multiple regressions for HOMA parameters were analyzed in pre-diabetic and normoglycemic subjects. 

## Results

 One-hundred sixty-three patients aged 10 to 31 years old who were suffered from beta-thalassemia major attended in this study. Of the 163 patients, 114 (69.93%) were male and 49 (30.07%) were female. The mean age was 17.80±5.27 years. Of the 163 participants with beta-thalassemia major, 10%, 53% and 37% were diabetic, pre-diabetic and normal cases, respectively. The patients’ characteristics related to blood sugar level are shown in Table 1. 

**Table 1:** Baseline characteristics of participants by blood glucose

**Parameters**	**Overall**	**Normal**	**Prediabetes**	**Diabetes mellitus**	**P value**
N (%)	163	61(37.42)	86(52.76)	16(9.82)	
Age (years)	17.80±5.27	17.08±5.59	18.06±5.33	19.62±4.53	0.15
Sex (%)					
Male	114 (69.93)	37(60.65)	59(68.60)	10 (62.50%)	0.04
Female	49 (30.07)	24(39.35)	27 (31.40)	6 (37.50%)	0.01
Height (cm)	150.59±12.67	149.96±14.04	153.96±11.27	144.83±12.26	0.05
Weight (kg)	40.05±10.30	40.07±12.23	41.78±10.43	36.16±7.22	0.19
Blood transfusion (U/month)	2.91±1.01	2.53±1.02	3.26±0.97	3.00±0.95	0.001
Duration of blood transfusion (years)	15.02±5.47	14.50±5.78	15.03±5.47	16.81±5.21	0.20
Ferritin (ng/ml)	4563.50±2849.30	2859.41±1528.73	3380.93±2955.56	4378.25±3314.00	0.03
Fasting plasma glucose (mg/dl)	121.93±72.14	92.52±17.92	116.88±19.65	294.37±152.32	0.0001
2h.post 75-g glucose (mg/dl)	156.52±43.97	149.43±56.48	164.68±19.80	208±38.76	0.35
Serum insulin level (µu/ml)	10.82±9.73	10.16±9.78	12.52±9.75	15.23±8.67	0.17
Serum zinc level (μg/dl)	18.90±10.93	20.15±12.55	19.46±11.29	17.43±6.67	0.91

Mean fasting serum glucose concentration was 92.52, 116.88 and 294.37 mg/dl in normal, pre-diabetic and diabetic participants, respectively. There was significant difference between ferritin and blood transfusion level. Mean serum zinc concentration was equal 18.90±10.93µg/dl which was not significantly different across diabetic, pre-diabetic and normal groups. It was found that insulin level was significantly different between normal and diabetic groups (P=0.02). No differences were found among the three groups with respect to weight, height, duration of blood transfusion, and 2h-post glucose load levels. Pre-diabetic patients had significantly lower beta-cell function in comparison with normal subjects (P=0.0001). However, no significant difference was found between serum zinc level, serum insulin, insulin sensitivity and insulin resistance across all three groups (Table 2). 

**Table 2:** Laboratory findings and Homeostasis Model Assessment (HOMA) parameters in normal and pre-diabetic subjects

**Parameter**	**Age**		**Blood transfusion**		**Serum ferritin**		**Duration of transfusion**	
	r	p	r	p	r	p	r	p
Fasting plasma glucose	0.08	0.23	0.12	0.13	0.11	0.19	0.08	0.24
Fasting Serum insulin	0.006	0.94	-0.15	0.14	-0.08	0.43	-0.07	0.38
Beta cell function	-0.04	0.57	-0.29	0.004	-0.27	0.03	-0.09	0.31
Insulin sensitivity	0.05	0.51	0.09	0.36	0.09	0.38	0.18	0.03
Insulin resistance	0.01	0.90	-0.14	0.17	-0.27	0.04	-0.08	0.37
Serum zinc level	-0.14	0.07	-0.06	0.46	-0.06	0.46	0.06	0.42

An inverse relationship was documented between the beta-cell function on the one hand and total units of blood transfusions and ferritin level on the other hand (r=-0.29, P=0.004). Beta-cell function correlated with Ferritin serum level (r=-0.27, P=0.03) and Insulin sensitivity correlated with duration of transfusion (r=0.18, P=0.03). An inverse relationship has also been seen between insulin resistance and serum ferritin level (r=-0.27, P=0.04). No correlation was seen between other parameters and age, total units of blood transfusion, serum ferritin and duration of transfusion (Table 3).

**Table 3:** Correlation between fasting plasma glucose, fasting plasma insulin, HOMA parameters, serum zinc level and other clinical and biochemical parameters

**Parameters**	**Normal glucose tolerance** **Mean±SD** **(Median)**	**Impaired glucose tolerance** **Mean±SD** **(Median)**	**Impaired fasting glucose** **Mean±SD** **(Median)**	**P value**
Fasting blood glucose (mg/dL)	92.52±17.92 (89.00)	100.76±4.85 (99.50)	145.55±82.08 (115.00)	0.0001
Serum insulin (µu/ml)	10.16±9.78 (9.45)	11.88±13.26 (6.40)	10.88 ±8.54 (7.90)	0.38
Serum zinc level (µg/dL)	20.15±12.55 (18.12)	19.67±7.73 (18.00)	19.31±11.32 (18.10)	0.93
Beta cell function (%)	119.82±72.30 (103.30)	94.88±71.09 (65.75)	65.59±40.04 (54.60)	0.0001
Insulin sensitivity(%)	106.22±62.02 (81.65)	113.92±62.97 (118.85)	119.63±72.21 (92.80)	0.66
Insulin resistance	1.41±1.23 (1.22)	1.53±1.62 (0.85)	1.37±1.12 (1.08)	0.75

As shown in Table 4, there was a neat correlation between beta-cell function and fasting plasma glucose as well as fasting serum insulin (P = 0.0001). In addition, insulin sensitivity and insulin resistance correlated with fasting serum insulin (P = 0.0001). No significant correlations were observed between other parameters.

**Table 4:** Correlation Between HOMA parameters and other biochemical parameters

**Parameter**	**Fasting blood glucose** **(mg/dL)**		**Fasting Serum insulin** **( µu/ml)**		**Serum zinc level** **( μg/dl)**	
	r	p	r	p	r	p
Beta cell function	-0.50	0.0001	0.74	0.0001	0.07	0.47
Insulin sensitivity	-0.01	0.88	-0.72	0.0001	-0.02	0.80
Insulin resistance	-0.08	0.33	0.99	0.0001	-0.04	0.70
Serum zinc level	-0.06	0.45	-0.02	0.78	1	

As shown in Table 5, some parameters were regressed against serum zinc levels. All parameters of HOMA were then regressed against serum zinc levels. Multiple linear regression analysis for HOMA parameters were carried out in normal subjects and participants with prediabetes (Table 6). After adjusting for multiple possible confounders in normal subjects and participants with prediabetes, this analysis did not show any significant association between HOMA parameters and serum zinc level.

**Table 5:** Adjusted linear regression for serum zinc levels in participants

**Parameters**	**Serum zinc**		
	**Coefficient**	**95% Confidence Interval**	**P value**
Glycemic status:			
Normoglycemic	Ref	Ref	Ref
Pre-diabetic	-7.66	-53.40 to 38.08	0.74
Diabetic	-26.67	-101.86 to 48.52	0.48
Age	-2.65	-7.45 to 2.13	0.27
Sex	-2.18	-46.85 to 42.48	0.92

**Table 6:** Adjusted linear regression analysis for HOMA parameters in normal and pre-diabetic subjets

	**HOMA2 Parameters**								
	**Beta Cell function**			**Insulin Sensitivity**			**Insulin Resistance**		
	Coefficient	95% CI	P value	Coefficient	95% CI	P value	Coefficient	95% CI	P value
Serum Zinc	0.16	-0.10-0.32	0.32	-0.02	-0.19-0.16	0.86	-0.09	-0.004- 0.002	0.57
Age	-0.24	-10.48-2.40	0.32	0.29	-1.39-9.36	0.14	-0.17	-0.14-0.05	0.38
Sex	0.01	-50.85- 56.60	0.91	0.005	-44.20- 45.56	0.97	0.06	-0.68-1.00	0.70

## Discussion

 In this study, we have shown high incidence of impaired glucose metabolism in beta-thalassemia major patients. Out of total 163 participants, 37% were normal, 53% were pre-diabetic and 10% were diabetes cases. The high prevalence of impaired glucose metabolism in the current study was in agreement with previous ones ^16^^-^^23^. Higher incidence of impaired glucose metabolism in our patients may be explained by the older age of our study group.

Worldwide about 60,000 children with a major thalassemia are born annually^24^. Employing better treatment approaches and improved chelation therapy, patients with thalassemia have longer life-expectancy. Therefore, new complications such as dysfunction of single or multiple endocrine glands are expected to occur. Diabetes mellitus is one of the most usual complications which appear gradually in the context of glucose metabolism disturbance among thalassemia major patients. The exact mechanism is unclear, but it may be the result of iron overload and pancreas haemochromatosis^25^. Moreover, Insulin resistance, liver dysfunction, genetic predisposition, family history of diabetes, lipid peroxidation, oxidative stress and release of free radicals are known predisposing factors^17^^, ^^26^. Glucose intolerance usually emerges during the second decade of life^8^. It seems that the earliest abnormality to be related to insulin resistance than to defect in insulin secretion. More effective iron-chelating therapy appears to improve glucose tolerance^27^.

Contrary to other studies, pre-diabetic patients in our survey showed significantly lower beta-cell function, but insulin level and insulin resistance index were not different between normal and pre-diabetic patients^12^. Our results showed both diabetic and pre-diabetic patients had higher fasting plasma insulin levels. 

The beta-cell function correlated significantly with iron overload indicators such as serum ferritin and total units of blood transfusion in our study groups. In a similar work, Mangiagli et al. showed similar findings.(Suvarna et al. 2006; Mangiagli et al. 1997) Zinc is an essential element in human body playing a significant role in many structures of enzymes. It is also a part of insulin crystal structure and is necessary for insulin production, storage, secretion, regulation of insulin receptor function and intracellular events involved in glucose tolerance and normal beta-cell reaction to glucose load ^18^^, ^^23^^, ^^29^^, ^^30^.

Antioxidant enzymes such as oxide desmutase, catalase and peroxidase require Zinc^31^, so it may protect insulin and beta-cells from free radicals by its antioxidant properties^19^. Previous studies have shown low level of serum zinc in patients with pre-diabetes and diabetes^32^^,^^33^. However, other studies did not find any difference in serum zinc concentration amongst the diabetic, pre-diabetic and normoglycemic groups^18^^, ^^34^. 

According to our study results, serum zinc level was not significantly different across diabetic, pre-diabetic and normal patients with beta thalassemia, but the level of zinc was low in all three groups. This is in the agreement with global zinc deficiency which affects almost 33% of world population^35^. Zinc deficiency could be related to poor nutrition, low-zinc-bioavailable diets like a diet rich in phytates and urinary loss in thalassemia patients just like normal population^36^^, ^^37^.

A strong point of our study is the large sample size. Patients at different ages were enrolled in this study. A potential limitation of this study is the absence of information on other important trace elements such as iron, copper and selenium which may influence serum zinc concentration^38^^,^^39^. Zinc deficiency in our patients was common and severe, and this affects the association of serum zinc level and HOMA parameters in three groups. Moreover, as this is a cross-sectional study, these results need to be replicated in longitudinal studies.

## CONCLUSION

 In conclusion, in agreement with other reports, our data on a population of patients with thalassemia major indicate that there is high prevalence of impaired glucose metabolism among patients with beta-thalassemia. This shows the importance of frequent follow-up and monitoring for early detection and appropriate therapy. There is no significant difference in the serum zinc concentration among normoglycemic, prediabetes, and diabetes groups.

Since the majority of our patients were zinc deficient, it was impossible to assess the effect of zinc status on glycemic status in patients with thalassemia. Further studies are required to confirm these findings, especially zinc level on glycemic status. Moreover, the results need to be replicated in longitudinal or cohort studies.
